# P-1892. Dabbling with Dalbavancin: Implementation and Cost-savings of an Inpatient-to-Outpatient Dalbavancin Pathway

**DOI:** 10.1093/ofid/ofae631.2053

**Published:** 2025-01-29

**Authors:** Melissa O’Neal, Nicholas Piccicacco, Kristen Zeitler, Jaimie Weber, Douglas McConnell, Chris Conn, Kari Carrieri, Susannah Hall, Jose Montero

**Affiliations:** Tampa General Hospital, Tampa, FL; Tampa General Hospital, Tampa, FL; Tampa General Hospital, Tampa, FL; Tampa General Hospital, University of South Florida Morsani College of Medicine, Tampa, Florida; Tampa General Hospital, University of South Florida Morsani College of Medicine, Tampa, Florida; Tampa General Hospital, Tampa, FL; Tampa General Hospital, Tampa, FL; Tampa General Hospital, Tampa, FL; University of South Florida Morsani College of Medicine, Tampa, FL

## Abstract

**Background:**

Dalbavancin is a lipoglycopeptide antibiotic with *in vitro* activity against gram-positive organisms and a terminal half-life of 14.4 days. One dose provides 7 days of therapy whereas a novel 2-dose regimen provides up to 4-6 weeks. These strategies are attractive alternatives to standard of care for patients in whom daily IV antibiotics are not feasible. There also may be a financial benefit as the dosing schedule allows for completion of therapy in the outpatient setting and can reduce hospitalization costs.

**Methods:**

This study is a retrospective chart review of all patients who received dalbavancin at Tampa General Hospital. Our institution approved the use of dalbavancin at no cost to patients for vancomycin-susceptible gram-positive infections who remain hospitalized only to receive IV antibiotics. All patients received at least one 1500mg dose inpatient to save 7 days of hospitalization; some patients planned to follow up a week later in an outpatient clinic for a second 1500mg dose to save at least 4 weeks of hospitalization. Cost estimates are based on the patient’s hospitalization cost prior to receiving dalbavancin and do not include avoided implicit costs.

**Results:**

From 12/13/22 to 3/31/24, 28 patients received dalbavancin; 14 (50%) planned to receive a second dose and 11 (78.6%) successfully received their dose in clinic. The majority of patients (60.7%) reported a history of injection drug use. Most infections were monomicrobial (85.7%) with *S. aureus* being the most common pathogen. An estimated 527 days of hospitalization were avoided, representing a cost savings of $684,712.44. The institutional drug cost for dalbavancin was $104,772, leading to an estimated net hospital cost savings of $579,940.44. The all-cause 30-day readmission or revisit rate was 28.6%, similar to reported readmission rates in this population, however the 30-day infection-related readmission or revisit rate was favorable (10.7%).

**Conclusion:**

The use of dalbavancin to facilitate discharge resulted in significant cost savings without increasing infection-related readmissions. Further cost-benefit analysis on true economic impact including the reduction in nosocomial infections and avoidance of toxicities is warranted.

**Disclosures:**

All Authors: No reported disclosures

